# The percentage abundance of sarcomatoid component has a prognostic role in grade 4 non-metastatic clear cell-renal carcinoma

**DOI:** 10.1007/s00345-025-05630-4

**Published:** 2025-04-23

**Authors:** Giuseppe Lucarelli, Francesco Lasorsa, Monica Rutigliano, Martina Milella, Marco Spilotros, Antonio d’Amati, Giuseppe Ingravallo, Felice Crocetto, Savio Domenico Pandolfo, Marco Fabiano, Matteo Ferro, Riccardo Autorino, Michele Battaglia, Pasquale Ditonno

**Affiliations:** 1 Urology, Andrology and Kidney Transplantation Unit, Department of Precision and Regenerative Medicine and Ionian Area, University of Bari “Aldo Moro”, Bari, Italy; 2 Pathology Unit, Department of Precision and Regenerative Medicine and Ionian Area, University of Bari “Aldo Moro”, Bari, Italy; 3 Department of Neurosciences, Reproductive Sciences and Odontostomatology, University of Naples Federico II, Naples, Italy; 4 Department of Urology, University of L’Aquila, L’Aquila, Italy; 5 Division of Urology, “Antonio Cardarelli” Hospital, Naples, Italy; 6 Urology Unit, Department of Health Science, University of Milan, Milan, Italy; 7 Department of Urology, Rush University Medical Center, Chicago, IL USA

**Keywords:** Renal cell carcinoma, Sarcomatoid, Grading, Percentage, Prognosis

## Abstract

**Purpose:**

Sarcomatoid dedifferentiation represents one of the most aggressive features of clear cell renal cell carcinoma (ccRCC). In this study we evaluated whether grade 4-ccRCC subclassification based on the intratumoral abundance of sarcomatoid features could have a prognostic impact.

**Methods:**

A cohort of 212 patients with localized or locally advanced sarcomatoid ccRCC was identified. This population was stratified according to abundance of sarcomatoid features in low-sarcomatoid (LS = < 20% sarcomatoid component; *n* = 117) and high-sarcomatoid (HS = ≥ 20% sarcomatoid component; *n* = 95). Estimates of cancer-specific survival (CSS) and recurrence-free survival (RFS) were calculated according to the Kaplan–Meier method and compared with the log-rank test. Multivariable analysis was performed using the Cox proportional hazards regression model to identify the most significant variables for predicting CSS and RFS.

**Results:**

Kaplan–Meier survival curves stratified by abundance of sarcomatoid component, showed that CSS and RFS were significantly decreased in patients with sarcomatoid component ≥ 20% (both *P* < 0.0001). At multivariable analysis by Cox regression modeling, the abundance of sarcomatoid component was an independent adverse prognostic factor for CSS (*P* < 0.0001) and RFS (*P* < 0.0001).

**Conclusion:**

ccRCC Subclassification based on the abundance of intratumoral sarcomatoid component has a clinical significance. Our study showed that ccRCC subclassification into HS versus LS groups had a prognostic impact in terms of CSS and RFS in non-metastatic ccRCC.

## Introduction

Renal cell carcinoma is one of the ten most frequent cancers worldwide, and it accounts for about 3–5% of all tumors. According to the American Cancer Society, 81,610 new cases and 14,390 kidney cancer deaths are projected to occur in the United States for 2024 [1].

Clear cell renal cell carcinoma (ccRCC) represents the most common malignant histotype, and according to 2022 (fifth edition) World Health Organization (WHO)/International Society of Urological Pathology (ISUP) grading system, 4 grades of progressive aggressiveness can be identified [2].

The actual 4-tier grading system updated the previous Fuhrman grade classification, and to date it represents an important prognostic parameter for risk stratification in patients with this cancer. The assessment of tumor grade is based on morphological features such as the degree of nucleolar prominence (for grade 1–3). An extreme nuclear pleomorphism and the presence of anaplastic giant cells, rhabdoid and/or sarcomatoid components describes the grade 4 [2].

Sarcomatoid dedifferentiation can occur in most RCC histologic subtypes, although it is most common in clear cell histotype, and identifies a highly aggressive phenotype associated with poor prognosis. In particular, the presence of sarcomatoid features is associated with higher stage at diagnosis, a more aggressive disease course, and reduced survival in both localized and metastatic settings [3].

Sarcomatoid RCC (sRCC) is not considered a distinct histological variant of RCC, but a pattern of dedifferentiation characterized by epithelial-mesenchymal transition (EMT) of epithelial component [4–6]. The cellular elements typically show a sarcoma-like spindle cell morphology with high pleomorphism and atypia and, according to WHO/ISUP criteria, the presence of any amount of these components is sufficient for the diagnosis of sRCC. Sarcomatoid RCC, like other tumors, is characterized by a marked phenotypic heterogeneity that ranges from a focal localization to extensive involvement of the sarcomatoid component. To date, few studies explored the role of the abundance of the sarcomatoid component in heterogeneous populations of patients with RCC [7–10].

The aim of this study was to evaluate the prognostic impact of the percentage abundance of the intratumor sarcomatoid features in a cohort of patients with non-metastatic RCC and with clear cell histology.

## Materials and methods

A cohort of patients with surgically resected localized or locally advanced sarcomatoid ccRCC (grade 4) was retrospectively evaluated from January 2016 to December 2023. Patients with visceral metastases at diagnosis were excluded. All patients were preoperatively staged by thoraco-abdominal Computed Tomography or Magnetic Resonance Imaging. Tumor staging was reassigned according to the ninth edition of the AJCC-UICC TNM classification.

Two dedicated genitourinary pathologists confirmed the presence of sarcomatoid feature in the neoplastic tissue, and the percentage of sarcomatoid component (PSC) was assessed on formalin-fixed paraffin embedded tissue blocks, without knowledge of patient outcome.

In particular, each tumor was entirely sampled and included for analysis. All histological slides were digitally scanned using a Nikon Hamamatsu NanoZoomer S60 scanner. The sarcomatoid areas were identified and selected on the digital scans with the assistance of QuPath software. This process was performed under the guidance of two experienced uropathologists, allowing for the precise assessment of the percentage of sarcomatoid areas for each slide and, ultimately, for each tumor case.

Tumor grade on pathological tissues was attributed with hematoxylin–eosin (HE) staining in according to 2022 WHO/ISUP grading system [2]. The concomitant presence of areas of ccRCC associated with the sarcomatoid component has always been identified. Clear cell RCCs with rhabdoid component were excluded.

**Fig. 1:**
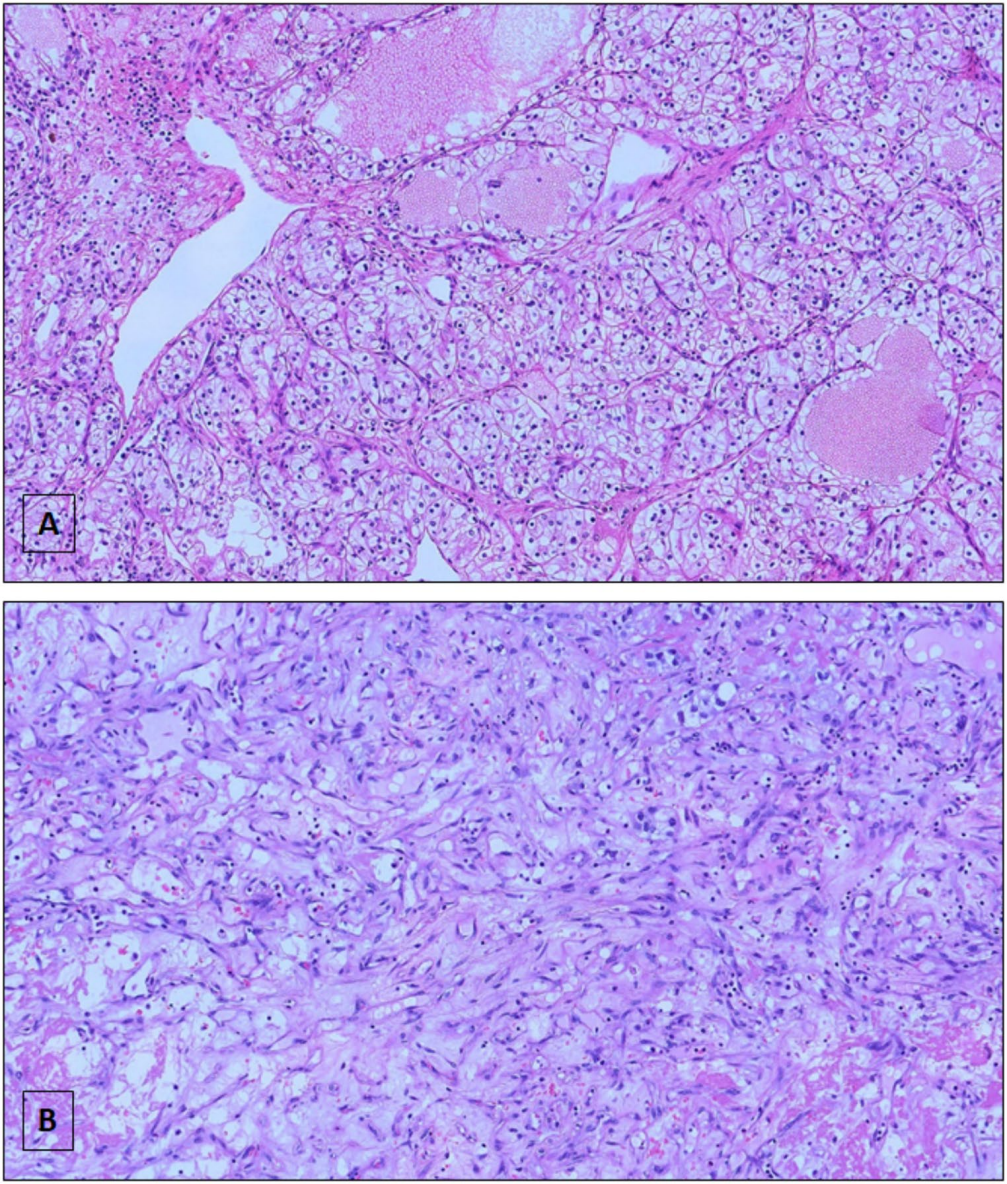
Hematoxylin–eosin staining of LS (**A**) and HS (**B**) ccRCC (10X). (LS = low sarcomatoid; <20% of sarcomatoid component). (HS = high sarcomatoid; ≥20% of sarcomatoid component)

Patients were stratified according to abundance of sarcomatoid features in low-sarcomatoid (LS: <20% of sarcomatoid component) and high-sarcomatoid (HS: ≥20% of sarcomatoid component) (Fig. 1A and B). The 20% cut-point was determined using receiver-operating characteristic (ROC) analysis and quantified in terms of area under the curve (AUC) and corresponding 95% confidence interval (95% CI). In particular, we used the event “death” and “recurrence” as binary markers, and identified the optimal cutoff value by intersecting the best sensitivity and specificity values.

Comparisons of median values between different groups were evaluated by Mann–Whitney U test. Continuous variables were summarized with median and 95% confidence interval for median (95% CI). Categorical variables were summarized with frequency counts and percentages.

Spearman test was applied to evaluate the correlations between the percentage of sarcomatoid component and tumor stage/size.

In the cancer-specific survival (CSS) analysis, patients who died of RCC-unrelated causes or were lost to follow-up were censored. Recurrence-free survival (RFS) was calculated from the date of surgery to the date of disease recurrence. Disease recurrence was assessed radiographically (using CT scan or MRI) with a surveillance schedule based on EAU guidelines. Estimates of CSS and RFS were calculated according to the Kaplan–Meier method and compared with the log-rank test. Multivariable analysis was performed using the Cox proportional hazards regression models to identify the most significant variables for predicting CSS and RFS. Sarcomatoid percentage was both used as a continuous or categorical variable. A backward selection procedure was performed with removal criterion *P* > 0.10 based on likelihood ratio tests. A P-value of < 0.05 was considered statistically significant.

Statistical tests were performed using MedCalc 19.6.3 (MedCalc software, Mariakerke, Belgium) and PASW 18 software (PASW 18, SPSS, Chicago, IL, USA).

Written informed consent to take part was given by all participants. The protocol for the research project has been approved by the local Ethics Committees and conforms to the provisions of the Declaration of Helsinki.

## Results

A total of 212 patients were included, of which 117 HS (55.2%) and 95 LS (44.8%). Most of the patients underwent radical nephrectomy (*n* = 184, 86.8%). No case of residual disease after nephrectomy was found. The analysis of global population, showed a male predominance and advanced T-stage in patients with sarcomatoid ccRCC (Table 1). ROC analysis for CSS identified a cut-off of 20% for sarcomatoid component (AUC = 0.61, 95%CI = 0.53–0.67; *P* = 0.006). Similarly, a cut-off of 20% was identified for RFS (AUC = 0.60, 95%CI = 0.51–0.69; *P* = 0.01).

**Table 1:** Clinical and pathological characteristics of patients with sarcomatoid CcRCC

	High Sarcomatoid (≥ 20%) *N* = 117	Low Sarcomatoid (< 20%) *N* = 95	*P*-value
Gender			
Male	72 (61.5%)	63 (66.3%)	0.5
Age at diagnosis			
Median 95% CI	62 57–64	64 58–67	0.03
**% Sarcomatoid Features**,** Median**	45%	10%	0.001
**Primary Tumor Size**,			
Median 95% CI	10 cm 7–8	8 cm 9–11	0.001
**Pathologic Tumor Stage**			
T1 T2 T3 T4	13 (11.2%) 15 (12.8%) 50 (42.7%) 39 (33.3%)	17 (17.9%) 18 (18.9%) 51 (53.7%) 9 (9.5%)	0.0006
**Pathologic Nodal Involvement**			
N0 N1	91 (78%) 26 (22%)	83 (87.4%) 12 (12.6%)	0.1
**Histology**			
Clear Cell	117 (100%)	95 (100%)	1.0
**First-Line Systemic Therapy**			
ICI-Based Combinations (ICBC) TKI (Excluding ICBC) No therapy	11 (9.4%) 101 (86.3%) 5 (4.3%)	12 (12.6%) 81 (85.3%) 2 (2.1%)	0.07
**Recurrence-Free Survival**			
Median 95%CI	11 months 10–14	32 months 18.5–35.8	< 0.0001
**Cancer-Specific Survival**			
Median 95%CI	16 months 15–17	41 months 35–45	< 0.001

ICI = Immune Checkpoint Inhibitor; TKI = Tyrosine Kinase Inhibitor

HS patients had larger masses than LS patients (*P* = 0.001). All patients were subjected to lymph node dissection and no statistically significant difference in nodal involvement between the two groups was found (*P* = 0.1).

Statistically significant differences resulted between the PSC and clinical stage (*P* = 0.003; Spearman correlation: rs = 0.23, *P* = 0.001), and tumor size (*P* < 0.0001; Spearman correlation: rs = 0.21, *P* = 0.004).

The median CSS was longer in LS group compared to HS (41 vs. 16 months, *P* < 0.0001). The median RFS was 32 months in LS and 11 months in HS patients (*P* < 0.0001).

**Fig. 2:**
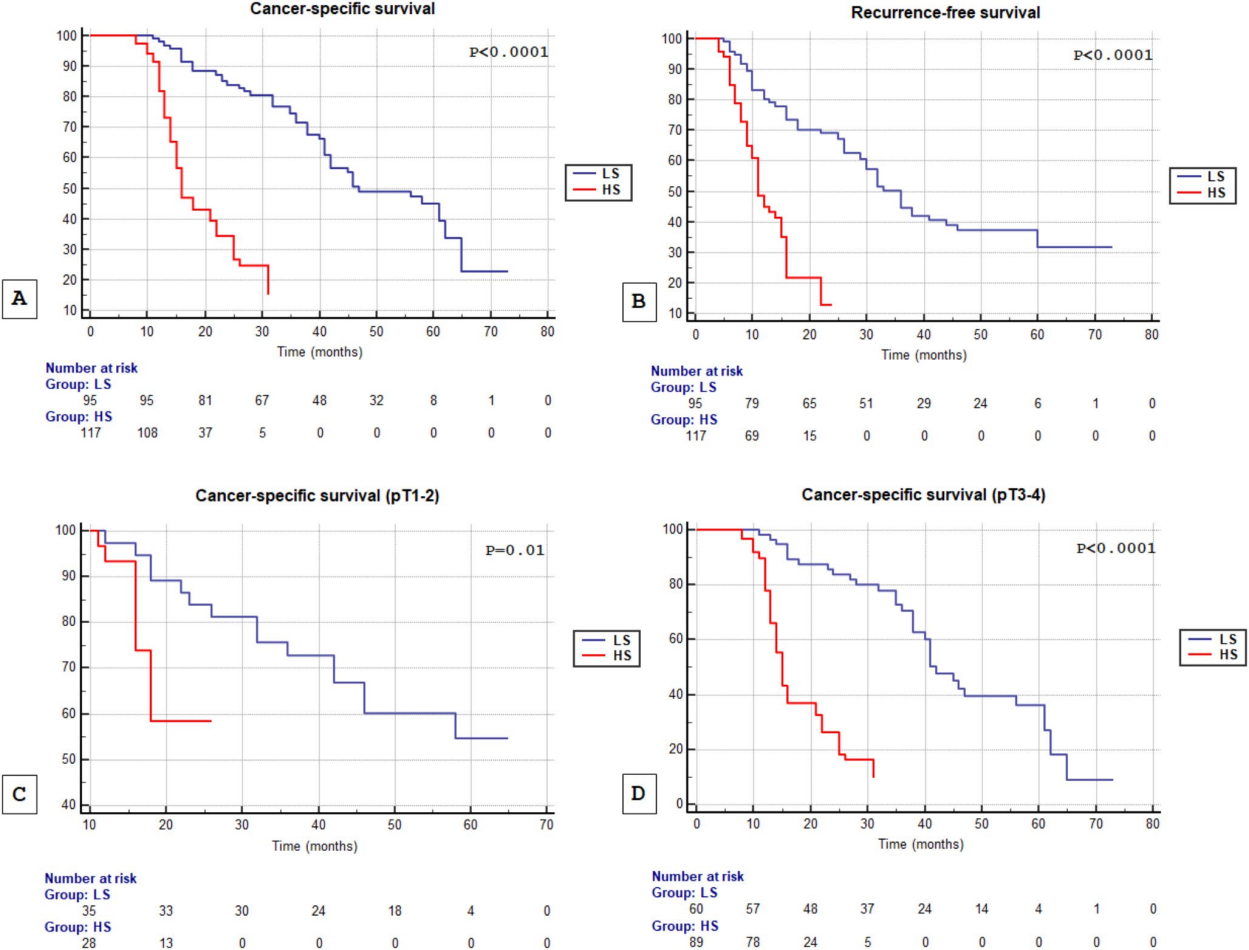
Kaplan-Meier cancer-specific survival (CSS) curves, stratified by abundance of sarcomatoid component (LS vs. HS) (**A**). Kaplan-Meier recurrence-free survival (RFS) curves, stratified by abundance of sarcomatoid component (LS vs. HS) (**B**). Kaplan-Meier cancer-specific survival (CSS) curves, stratified by abundance of sarcomatoid component (LS vs. HS), in pT1-2 patient population (**C**). Kaplan-Meier cancer-specific survival (CSS) curves, stratified by abundance of sarcomatoid component (LS vs. HS), in pT3-4 patient population (**D**)

Kaplan–Meier survival curves stratified by abundance of sarcomatoid component, showed that CSS and RFS were significantly decreased in patients with sarcomatoid component ≥ 20% (both *P* < 0.0001) (Fig. 2A and B). When patients were stratified according to pathological stage (pT1-2 and pT3-4), PSC ≥ 20% was associated with higher risk of death (Fig. 2C and D) (*P* = 0.01 and *P* < 0.0001, respectively).

At multivariable analysis by Cox regression modeling, including PSC, tumor size, age, T and N stage as predefined variables, the abundance of sarcomatoid component when evaluated as a continuous or categorical variable, was an independent adverse prognostic factor for CSS (HR = 1.03, *P* < 0.0001; and HR = 3.36, *P* < 0.0001, respectively) and RFS (HR = 1.01, *P* = 0.0001; and HR = 2.82, *P* = 0.0001, respectively) (Table 2).

**Table 2:** Cox multivariable linear regression models for survival using sarcomatoid percentage as a continuous variable and sarcomatoid percentage as a categorical variable (< 20%, ≥ 20%)

**Cancer-specific Survival - Sarcomatoid Percentage as a Continuous Variable**			
**Variable**	**Value**	**Hazard Ratio (95%CI)**	***p***-**value**
Sarcomatoid Percentage	Continuous	1.03 (1.01–1.04)	< 0.0001
Tumor Size (cm)	Continuous	1.02 (0.98–1.06)	0.08
T Stage	3–4 vs. 1–2	1.51 (1.18- 92)	0.001
N Stage	N + vs. N0	0.65 (0.41–1.06)	0.08
Age	Continuous	0.98 (0.95–1.00)	0.07
**Recurrence-free Survival - Sarcomatoid Percentage as a Continuous Variable**			
**Variable**	**Value**	**Hazard Ratio (95%CI)**	**p-value**
Sarcomatoid Percentage	Continuous	1.01 (1.0–1.02)	0.0001
Tumor Size (cm)	Continuous	1.06 (1.01–1.12)	0.07
T Stage	3–4 vs. 1–2	2.26 (1.48–3.49)	0.001
N Stage	N + vs. N0	0.95 (0.65–1.41)	0.09
Age	Continuous	0.98 (0.96–1.18)	0.09
**Cancer-specific Survival - Sarcomatoid Percentage as a Categorical Variable**			
**Variable**	**Value**	**Hazard Ratio (95%CI)**	**p-value**
Sarcomatoid Percentage	High (≥ 20%) vs. Low (< 20%)	3.36 (1.64–6.81)	< 0.0001
Tumor Size (cm)	Continuous	1.02 (0.98–1.07)	0.09
T Stage	3–4 vs. 1–2	2.63 (1.67–4.13)	0.001
N Stage	N + vs. N0	1.01 (0.97–1.24)	0.07
Age	Continuous	0.97 (0.94–1.00)	0.09
**Recurrence-free Survival - Sarcomatoid Percentage as a Categorical Variable**			
**Variable**	**Value**	**Hazard Ratio (95%CI)**	**p-value**
Sarcomatoid Percentage	High (≥ 20%) vs. Low (20%)	2.82 (1.98–5.82)	0.0001
Tumor Size (cm)	Continuous	0.98 (0.94–1.08)	0.07
T Stage	3–4 vs. 1–2	2.27 (1.53–3.56)	0.001
N Stage	N + vs. N0	0.78 (0.53–1.15)	0.1
Age	Continuous	0.97 (0.95–1.01)	0.08

## Discussion

The prognosis of sRCC is poor and in about 60–80% of cases this tumor is diagnosed in locally advanced or metastatic stage [3].

The scarcity of data on this aggressive variant of RCC highlights the urgent need to acquire additional information about the molecular mechanisms underlying its development, and to identify specific biomarkers for diagnostic purposes and risk assessment. In recent years, the use of high-throughput platforms has provided novel evidence that sRCC is characterized by a distinct molecular pathogenesis, and different mutational and transcriptional profiles compared to non-sRCC [4, 5].

The recent findings of an integrative molecular characterization of sRCC, showed that this tumor harbor distinctive molecular features including BAP1, CDKN2A, and Hippo pathway mutations. Moreover, sRCC exhibited an increased expression of MYC-regulated transcriptional programs and an immune-inflamed phenotype highly responsive to immune checkpoint inhibitors (ICIs) [5, 6].

Previous retrospective studies have shown reduced CSS in relation to the percentage of sarcomatoid component. However, these reports evaluated patients with different histologic subtypes and included both metastatic and non-metastatic cases. Abidi et al. [7], evaluated for the first time the prognostic role of PSC on overall survival of patients with localized and metastatic sRCC. These authors used a cutpoint of 10% for patients stratification and found that subjects with PSC > 10% had reduced OS compared to patients with PSC ≤ 10% (*P* = 0.04).

Another retrospective study found in a small cohort of patients with sRCC that a PSC > 25% was an independent predictor of poor OS in a non-metastatic setting (HR = 2.07, *P* = 0.48) [8]. However, this study showed that PSC had no prognostic role in metastatic disease. Zhang et al. [9] showed that in patients with grade 4 RCC with or without visceral metastases at diagnosis, sarcomatoid differentiation was associated with adverse survival in a multivariable model (HR = 1.58, *P* < 0.001). In addition, this study demonstrated that the abundance of sarcomatoid component (PSC cut-off = 30%) was an independent prognostic factor for CSS (HR = 1.06, *P* = 0.028).

More recently, a study explored the prognostic role and the biological basis of sarcomatoid heterogeneity in a cohort of patients with both localized and metastatic sRCC. When analyzed as a continuous variable, PSC was associated with reduced OS (HR = 1.02, *P* < 0.001) and metastasis free survival (HR = 1.02, *P* = 0.02) [9].

To better define the molecular characteristics underlying the sarcomatoid heterogeneity, bulk RNA sequencing and GSEA [11] were performed in HS and LS RCC (PSC cutoff = 10%) [10]. c-Myc targets, EMT program, and mTOR pathway were significantly enriched in HS vs. LS tumors. In addition, HS RCC showed enrichment in complement system activation [12–14] and in different metabolic processes including glycolysis and fatty acid metabolism [15, 16]. Conversely, LS tumors were enriched for hypoxia-dependent signaling, oxidative phosphorylation, and angiogenesis.

Our study evaluated whether grade 4 RCC subclassification based on the intratumoral abundance of sarcomatoid features could have a prognostic implication using a cutpoint of 20% for PSC. Compared to previous studies, we enrolled only patients with non-metastatic disease and with clear cell histology. Our results showed that for all patients with grade 4 RCC, the presence of sarcomatoid component was associated with an increased risk of death and recurrence. In particular, we classified our population on the basis of the intratumoral abundance of sarcomatoid component using a 20% cutoff. Kaplan–Meier survival curves showed that CSS and RFS were significantly decreased in patients with sarcomatoid component ≥ 20% and these differences were confirmed also in subgroup analyses for pathological stage. Multivariate analysis confirmed the role of intratumoral abundance of sarcomatoid component as independent risk factor for CSS and RFS.

The main limitations of this study include its retrospective design, the lack of external validation and some variability among pathologists in evaluating the PSC.

Sarcomatoid RCCs are responsive to ICIs [17, 18] and are characterized by immune system activation, increased CD8^+^ T cell infiltration and PD-L1 expression [5, 19]. Unfortunately, in our study the number of patients treated with adjuvant ICIs was too low for an unbiased statistical analysis. Thus, additional investigations to evaluated the impact of PSC on ICIs responsiveness are mandatory.

## Conclusions

Sarcomatoid ccRCC is a highly aggressive form of renal cancer. Our study showed that ccRCC subclassification into HS versus LS groups had a prognostic impact in terms of CSS and RFS in non-metastatic ccRCC. Subclassification based on the abundance of intratumoral sarcomatoid component has a clinical significance and its pathological evaluation should be routinely performed.

The integration of this data into clinical nomograms can improve the prognostic information provided by these tools.

## Data Availability

No datasets were generated or analysed during the current study.
